# A Rare Case of Primary Nasal Tuberculosis

**DOI:** 10.7759/cureus.101214

**Published:** 2026-01-10

**Authors:** Khushboo G Malhotra, Ajeet K Khilnani, Ayush Tank, Aabha Parmar, Preet Hingrajiya

**Affiliations:** 1 Otolaryngology-Head and Neck Surgery, Gujarat Adani Institute of Medical Sciences, Bhuj, IND

**Keywords:** acid-fast bacilli, anti-tubercular therapy (att), diagnostic nasal endoscopy, nasal septal perforation, primary nasal tuberculosis

## Abstract

Primary nasal tuberculosis (TB) is an exceedingly rare form of extrapulmonary TB, often mimicking other granulomatous diseases and posing diagnostic challenges. We present a case of a 54-year-old female patient with persistent nasal obstruction and epistaxis, initially suspected to be chronic rhinosinusitis. Diagnostic evaluation, including nasal endoscopy and biopsy, revealed caseating granulomas with acid-fast bacilli (AFB), confirming primary nasal TB without pulmonary involvement. The patient was treated with a standard anti-tubercular regimen of two months of intensive phase (four drugs) and four months of continuation phase (three drugs), resulting in complete resolution of symptoms and no recurrence at one-year follow-up. This case underscores the importance of considering TB in atypical nasal presentations, especially in endemic regions, and highlights the efficacy of timely anti-tuberculous therapy.

## Introduction

Tuberculosis (TB) remains a global health challenge, primarily affecting the lungs but capable of involving extrapulmonary sites in approximately 15-20% of cases. [1] Nasal TB, a subset of extrapulmonary TB, is exceptionally rare, accounting for less than 1% of all TB cases and even fewer as primary manifestations without evidence of lung disease [1]. The first documented case of nasal TB dates back to 1761, reported by Giovanni Morgagni during an autopsy [2]. People with low immunity, residing especially in the high-incidence areas, are vulnerable to primary upper respiratory TB [3]. The most common presenting symptom of nasal TB is nasal obstruction, followed by nasal discharge, nasal bleed, crusting, eye watering, postnasal discharge, recurrent nasal polyps, and ulceration, which can lead to misdiagnosis as conditions like granulomatosis with polyangiitis (GPA), sarcoidosis, or malignancy [4]. Diagnosis relies on histopathological evidence of granulomatous inflammation, acid-fast bacilli (AFB) staining, culture, or molecular tests like polymerase chain reaction (PCR) [5]. We report a rare case of primary nasal TB to illustrate its clinical features, diagnostic approach, and management.

## Case presentation

A 54-year-old female patient presented to the otolaryngology clinic with a three-month history of progressive left-sided nasal obstruction, intermittent epistaxis, and mucopurulent nasal discharge. She denied fever, weight loss, cough, or night sweats, and had no history of TB exposure, immunodeficiency, or recent trauma. Her medical history was unremarkable, and she was a nonsmoker with no occupational risk factors.

Anterior rhinoscopy examination revealed a polypoidal mass in the left nasal cavity with edematous mucosa and whitish granulomatous patches on the nasal septum. There was no external nasal deformity, cervical lymphadenopathy, or ear involvement. The chest X-ray was normal, ruling out pulmonary TB.

**Figure 1:**
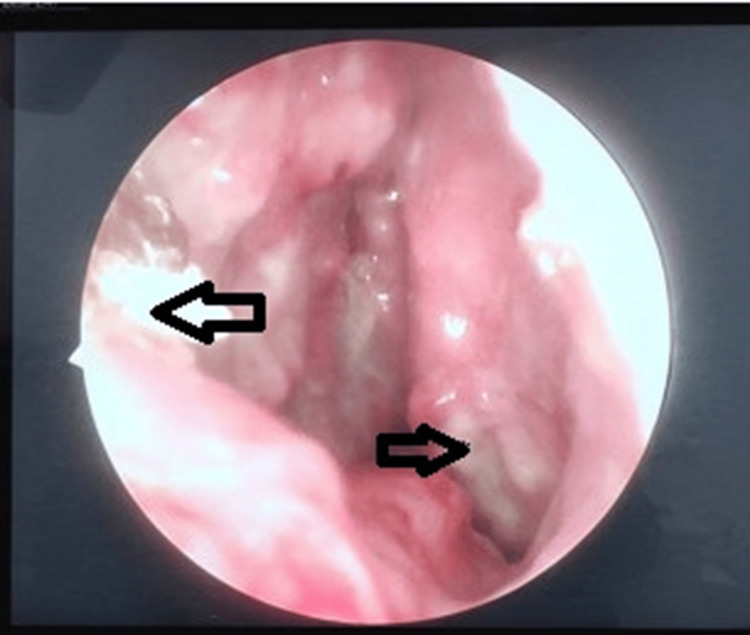
Left nasal endoscopic view showing ulcerative lesion over nasal septum and inferior turbinate (black arrows)

Nasal endoscopic examination showed friable, ulcerated lesions on the left nasal septum and the inferior turbinate, with surrounding pale mucosa (Figure 1). The lesions were pale and painless and did not bleed on touch.

**Figure 2:**
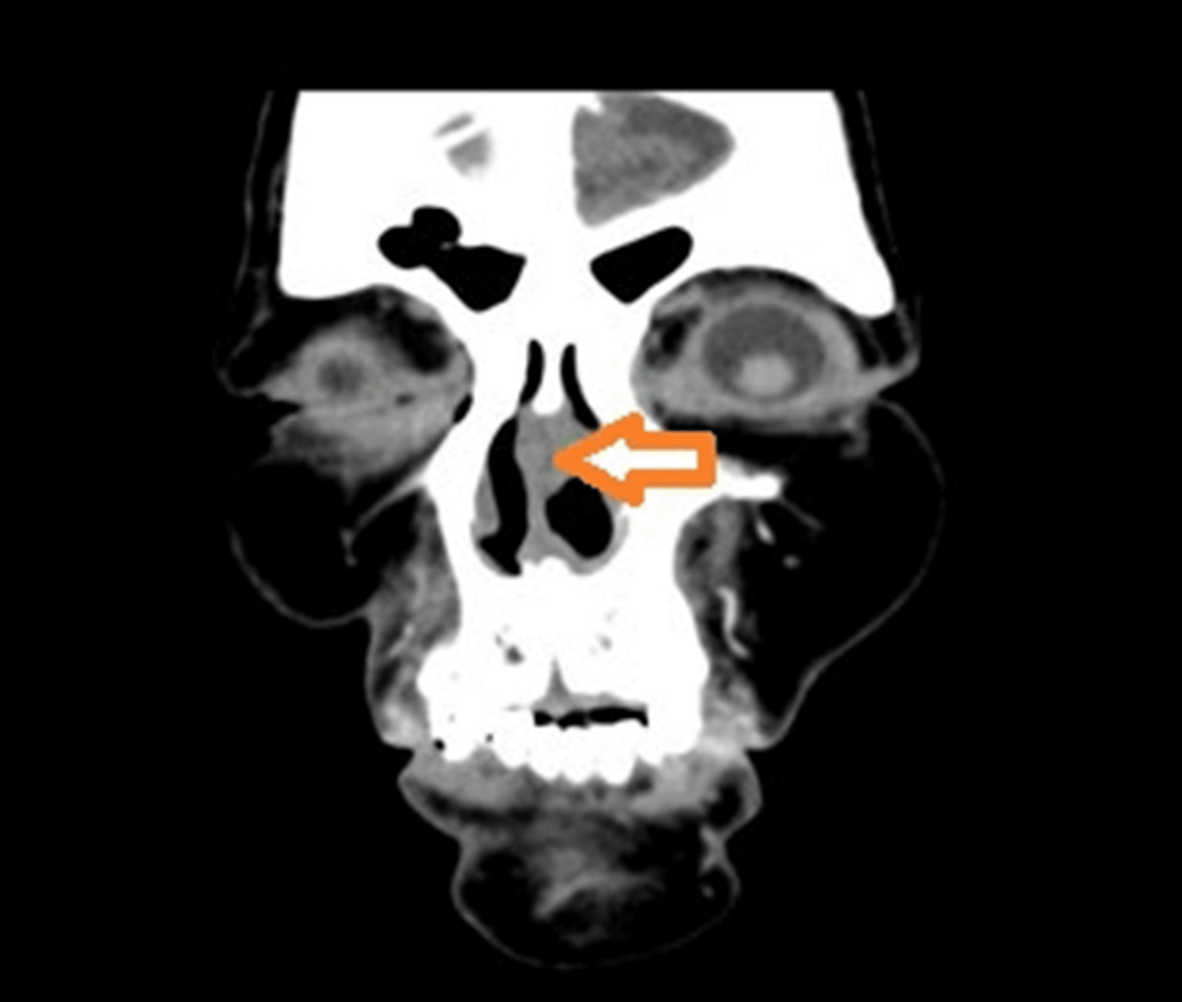
CT PNS showing soft tissue opacification of left side nasal septum (orange arrow) CT: computed tomography; PNS: paranasal sinuses

Computed tomography (CT) of the paranasal sinuses demonstrated soft tissue opacification involving the cartilaginous part of the left side of the nasal septum (Figure 2). The paranasal sinuses were not involved.

**Figure 3:**
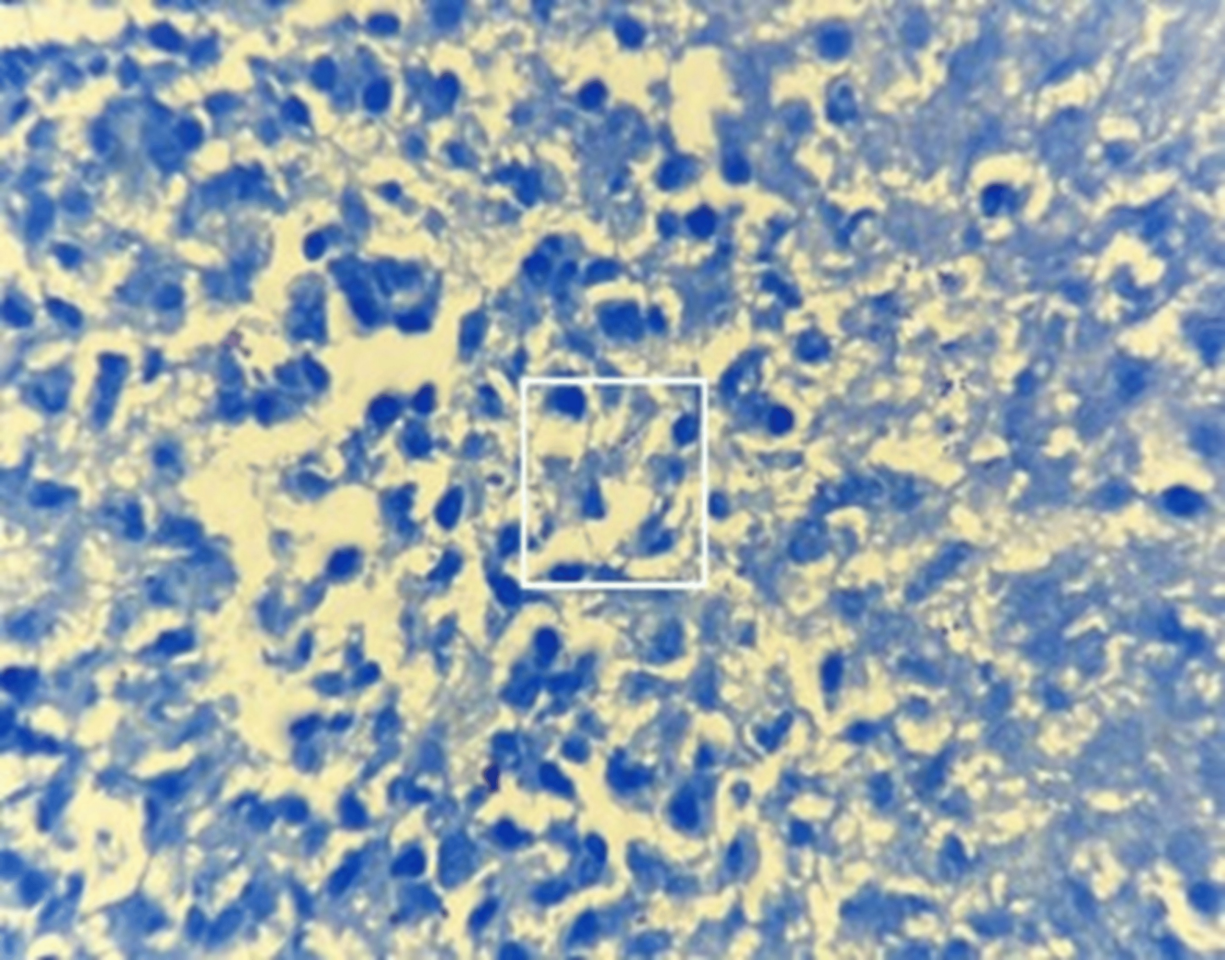
ZN stain (x 100) showing acid fast bacilli (white square) ZN: Ziehl-Neelsen

Biopsy of the nasal lesion was performed, and histopathological examination (HPE) revealed polypoidal nasal tissue comprising marked chronic inflammation, an area of necrosis, and necrotic debris. There was no evidence of granuloma or malignancy. Ziehl-Neelsen (ZN) staining demonstrated AFB (Figure 3). 

**Figure 4:**
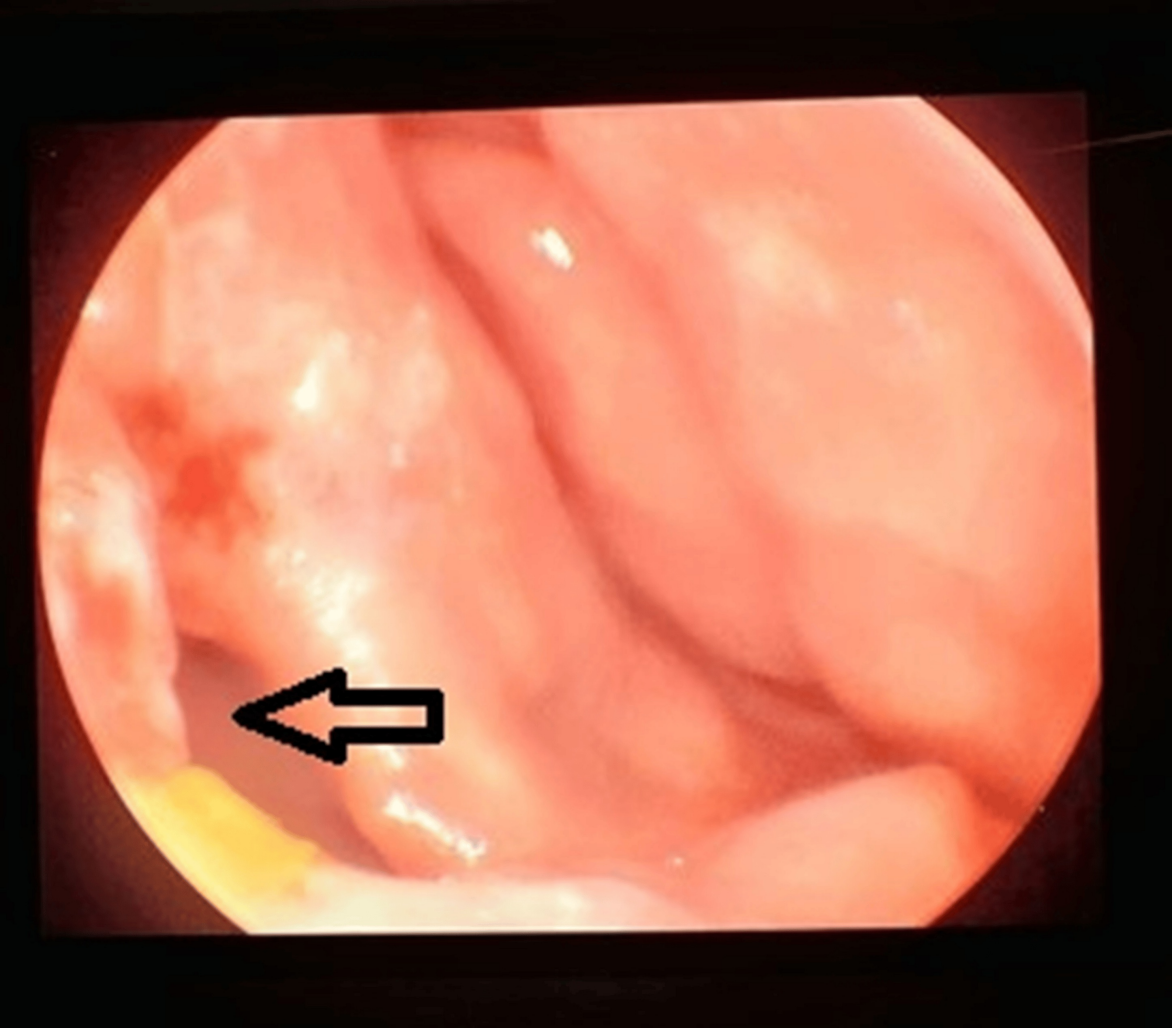
Left nasal endoscopic view showing resolution of lesions and a septal perforation (black arrow)

Based on the above work-up, the patient was diagnosed with a case of primary nasal TB. Four-drug anti-tubercular treatment (isoniazid (INH), rifampicin (RIF), pyrazinamide (PZA), and ethambutol (EMB)) was started. The patient took the chemotherapy for six months (two months of intensive phase (four drugs) and four months of continuation phase (three drugs)), resulting in complete resolution of symptoms and no recurrence at one-year follow-up. The patient, however, had an asymptomatic nasal septal perforation (Figure 4). 

Granulomatous disease, such as TB, affects the cartilaginous part of the nasal septum, whereas syphilis affects the bony part of the nasal septum. Septal perforation, a known sequela for TB, was found in our patient post completion of anti-tubercular treatment, but as the patient was asymptomatic, no further management was done for the same, and the patient is kept for regular follow-up.

## Discussion

Primary TB of the nose is exceedingly rare, with fewer than 100 cases having been reported in the literature. There is a distinct female preponderance (female-to-male ratio is 3:1), and the mean age at diagnosis has been reported to be 40 years [6]. The nasal septum is more frequently involved than the lateral wall; if the latter is affected, the inferior turbinate is the most common location [7]. In endemic areas, the resurgence of extrapulmonary TB may be linked to factors like human immunodeficiency virus (HIV) co-infection or immunosuppressive therapies such as anti-tumor necrosis factor (TNF) agents, which can reactivate latent TB [1]. The diagnostic dilemma arises as the clinical features mimic other more common nasal pathologies [6]. For instance, GPA often presents with similar nasal crusting and ulceration but is distinguished by granuloma formation (a collection of histiocytes made up of modified macrophages/epithelioid cells), often surrounded by T lymphocytes and multinucleated giant cells [8]. Sarcoidosis features noncaseating granulomas, while fungal infections or malignancies require specific cultures or immunohistochemistry. In our patient, the absence of systemic symptoms and normal chest imaging supported a primary nasal focus, consistent with reported cases where pulmonary involvement is absent. 

Treatment follows standard guidelines for extrapulmonary TB, typically involving a six- to nine-month regimen of INH, RIF, PZA, and EMB in the intensive phase, followed by INH, RIF, and EMB in the continuation phase [9]. In our case, the patient took the chemotherapy for six months (two months of intensive phase (four drugs) and four months of continuation phase (three drugs)), resulting in complete resolution of symptoms. Among the treatment options for nasal TB are drug therapy, surgical excision, diathermy, cautery, and radiotherapy [6]. Outcomes are favorable with early diagnosis, as evidenced by symptom resolution and low recurrence rates in follow-up [8].

## Conclusions

This case highlights the need for a high index of suspicion for nasal TB in patients with chronic nasal symptoms unresponsive to conventional therapy. Prompt biopsy and microbiological confirmation are crucial to differentiate it from mimickers and initiate effective anti-tuberculous treatment, leading to an excellent prognosis. Increased awareness may reveal more cases, potentially indicating a resurgence rather than mere coincidence in reporting.
